# A comparison of soil texture measurements using mid-infrared spectroscopy (MIRS) and laser diffraction analysis (LDA) in diverse soils

**DOI:** 10.1038/s41598-020-79618-y

**Published:** 2021-01-08

**Authors:** Cathy L. Thomas, Javier Hernandez-Allica, Sarah J. Dunham, Steve P. McGrath, Stephan M. Haefele

**Affiliations:** Rothamsted Research, West Common, Harpenden, Hertfordshire AL5 2JQ UK

**Keywords:** Environmental chemistry, Infrared spectroscopy, Optical spectroscopy

## Abstract

Spectroscopic methods for the determination of soil texture are faster and cheaper than the standard methods, but how do the results compare? To address this question, laser diffraction analysis (LDA) and mid-infrared spectroscopy (MIRS) analysis have been compared to conventional sieve-pipette measurements of texture in diverse European and Kenyan soils. To our knowledge this comparison between LDA and MIRS has not been made previously. It has used soils with a broad range of organic carbon (OC) contents to investigate whether, as in other techniques, clay-OC aggregation affects the estimation of clay with MIRS. The MIRS predictions of clay content were much better than the LDA measurements, but both techniques gave good measurements of sand content. The MIRS over-estimated clay at low clay content and under-estimated at high clay content (calibration set R^2^ = 0.83). The LDA over-estimated clay by ~ 60% (calibration set R^2^ = 0.36), indicating that the widely used clay threshold of < 8 µm was too high, and < 4 µm was found to be more accurate. In samples with < 5% OC content, both the LDA and MIRS gave very good clay predictions (R^2^ = 0.88 and 0.81, respectively). But in predictions of clay content in samples with > 5% OC the LDA under-estimated (R^2^ =  < 0.1) and MIRS over-estimated (R^2^ = 0.34) clay content. In soils with OC removed, the MIRS prediction of clay content improved, indicating interference between over-lapping spectral regions for organic and mineral constituents. Unlike granulometric measurements of texture such as the LDA, MIRS analysis is not subject to the limitations imposed by the shape and density of particles. It was concluded that in typical agricultural soils with < 5% OC and < 60% clay content, both techniques could be used for cheap, fast and reliable estimates of soil texture.

## Introduction

Soil texture, describing the relative proportion of sand, silt and clay in the mineral phase of soils is a major determinant of its water storage capacity and permeability, aeration, bulk density, aggregate stability and carbon storage capacity. Clay particles have high cation exchange capacity which affects nutrient availability to crops. Thus, knowing the texture of a soil is essential to understanding how well it functions for crop production and other soil functions.

Conventional measurements of soil texture use the sieve-pipette and hydrometer^1^ techniques, which are gravitational-sedimentation methods and make granulometric measurements of grain size. In the sieve-pipette method, the clay fractions (< 2 µm) are measured by sampling from a soil in solution using a pipette and calculating the dry weight, this is done after the sand fraction (63–2000 µm) has been collected with sieving. The hydrometer method measures the clay fraction from the density of the soil in water using the principle of buoyancy, and the sand fraction is often also collected with sieving. Both are based on the assumptions of Stokes’ law; particles have a constant bulk density and all particles of a particular density will settle at a certain rate. This law also assumes a homogeneous particle shape i.e. a spherical particle shape, which is more applicable to sand than to clay particles. Non-spherical particles have higher resistance than a sphere and thus a reduced settling velocity, leading to an under-estimate of the size of non-spherical clay particles and an over-estimation of the smallest clay proportion^2^. Similarly, sieving can allow non-spherical silt/sand particles to pass through the sieve on the smaller edge and thus over-estimate the proportion of the clay fraction^3^. Thus, shape effects are accentuated for non-spherical particles most often found with the clay fraction. For example, it has been found that compared to absolute particle size measurements made using an electron microscope, the sieve-pipette method significantly over-estimated clay content by including particles up to 5 µm diameter^4^.

Laser diffraction analysis (LDA) is a high-throughput spectroscopic method for texture analysis, which uses forward scattering of monochromatic coherent light; the angle of diffraction of light off a particle is inversely related to the size of the particle. Like the standard techniques, laser diffraction analysis is also a granulometric approach, which whilst not making assumptions about settling velocity, it does also assume sphericity of particles; that the cross-sectional area of one surface applies to all surfaces, and this assumption leads to clay under-estimation, because the large platy surface area is interpreted as sphere shape and hence a larger particle size^3–7^. This particle shape effect was demonstrated when the size of pure quartz samples milled to < 2 µm were measured very accurately by LDA, whereas the size of true clay samples < 2 µm were significantly over-estimated^8^. Therefore, the conventional technique described above tends to over-estimate the clay fraction, whereas the LDA tends to under-estimate it.

Furthermore, the basis of granulometric measurements is the dispersion of soil aggregates into individual particles by chemical, mechanical or ultrasonic means. However, often the soil aggregates are not easily dispersed, and pre-treatment to remove soil organic matter (SOM), Fe oxides and carbonates which bind particles are required^2,9^. It has been found that in soils with > 2% SOM, clay will be under-estimated using the sieve-pipette method because of aggregation^5^ or the hydrometer method^10^. This has also been observed with the LDA technique^7,11^.

Another disadvantage of gravitational-sedimentation techniques is that they are extremely time-consuming. Other more high-throughput spectroscopic methods, in addition to LDA, include; X-ray attenuation^12^, Coulter/electrical sensing zone method, and infrared spectroscopy with visible (vis), near-infrared (NIR) and mid-infrared (MIRS) radiation. Unlike the afore mentioned techniques, infrared analyses the chemistry of soil constituents. Mid-infrared spectroscopy is sensitive to both organic and inorganic phases, so is ideal for soils. Fundamental molecular frequencies occur in the MIRS region between 600–4000 cm^−1^, such as C-H and C-N bonds in organic materials and Si–O bonds in minerals. Overtones and combinations of these fundamental frequencies occur in the NIR region, making quantification in the NIR region more difficult^13^. Mid-infrared spectroscopy has proved successful at determining multiple soil properties, including texture^13–18^. These high-throughput spectroscopic techniques are also more economical and have the potential to give estimations of multiple soil properties, therefore allowing a higher density of soil sampling in large field surveys, or within fields, and facilitating a precision agriculture approach.

The objective of this study was to assess how texture measurements made by the LDA and MIRS compare to the reference sieve-pipette method. Diverse soil sets with a broad range of texture and OC content were studied to allow comparison of how the methods perform for different soil types. The study also used soils with OC destroyed to investigate whether clay-OC aggregation affects the estimation of clay with MIRS, as it has previously been shown to do with other techniques. It was hypothesised that MIRS analysis would give better predictions than the LDA of clay, because is it not subject to the limitations imposed by the shape and density of non-spherical clay particles. However, it was uncertain as to whether the MIRS technique was also affected by high OC content causing the aggregation and occlusion of clay particles and preventing detection.

## Materials and methods

### Soil sample sets

European soils were selected from the Rothamsted Research soil archive based on known soil texture and organic carbon contents to cover a very diverse range of soil types (n = 75). The soils were from 11 countries, with the majority being from England (n = 55). The clay content was 1.5–57%, sand content was 8–95% and OC content was < 1–33%. The European set was further split into sets with < 5% OC (organic carbon, the majority with < 3% OC) and > 5% OC, to investigate the effect of OC on texture measurements. The independent validation sets are from arable fields across the UK and were also selected from the archive, to test whether the narrower range of texture could be better predicted in these sets. The first independent validation set were soils from plots of the Broadbalk field trial (Rothamsted Research, UK), which is a long-running field experiment receiving different rates of fertiliser and manure (n = 46); clay content was 20–39%, sand + silt content was 61–80% and OC content was 0.8–3.5%. A second independent validation set contained diverse soils from arable fields across the UK (n = 25); clay content was 4–59%, sand content was 5–92% and OC content was 1.1–14% (Table 1; raw Supplementary Information, Appendix A).

**Table 1:** Mean, minimum and maximum of soil clay and sand measured using the sieve-pipette method, and organic carbon (OC, %), in all European and Kenyan soil sets. Fold difference = maximum/minimum value. Cal. = calibration, val. = validation.

	Min (%)	Mean (%)	Max (%)	Fold-difference
**European sets**				
European whole cal. set (n = 75)				
Clay (< 2 µm)	1.5	27	58	39
Sand (> 63 µm)	4	39	95	24
OC (%)	0.3	4.3	33	110
Broadbalk field val. set (n = 46)				
Clay (< 2 µm)	20	27	39	2
Silt + Sand (> 2 µm)	61	74	80	1
OC (%)	0.8	1.4	3.5	4
UK val. set (n = 23)				
Clay (< 2 µm)	4	25	59	15
Sand (> 63 µm)	5	46	92	18
OC (%)	1.1	3.4	14	13
**Kenyan sets**				
Kenya cal. set (n = 16)				
Clay (< 2 µm)	3	34	59	20
Sand (> 63 µm)	6	45	96	16
OC (%)	< 0.1	1.9	5.7	57
Kenya farms val. set (n = 30)				
Clay (< 2 µm)	9	28	48	5
Sand (> 63 µm)	36	56	77	2
OC (%)	0.6	1.4	2	3

For the Kenyan soil texture calibration, the ICRAF (World Agroforestry Centre) in-house standards (n = 16) were used, which are a diverse set of Kenyan soils which had been selected for diversity from the ICRAF soil archive based on MIRS spectra using the Kennard-Stone algorithm. The clay content was 3–59%, sand content was 6–96% and OC content was < 1–5.7%. The independent validation set soils were from small-holder farms in Bungoma county, western Kenya (n = 30), which had been selected for diversity from a wider sample set of small-holder farm soils based on MIRS spectra using the Kennard-Stone algorithm; clay content was 9–48%, sand content was 36–77% and OC content was < 1–2% (Table 1; raw Supplementary Information, Appendix A).

All soils were air-dried and sieved to < 2 mm. A sub-sample of the soil was ground to powder (< 50 µm) for 5 min at 700 RPM in a Retsch PM400 all-agate planetary ball mill (Retsch GmbH, Germany) for the MIRS analysis. The OC of all samples was also analysed: total carbon was analysed with a LECO TruMac Combustion Analyser (LECO, Michigan, USA), and inorganic carbon was analysed using combustion with a Skalar Primacs (Skalar Analytical BV, Breda, Netherlands), and organic carbon was calculated from total carbon minus inorganic carbon.

### Texture analysis using sieve-pipette (reference method)

Of each of the soil samples, one replicate of 100 g of < 2 mm soil was analysed using the pipette- sedimentation with sand fractionation method (ISO 11277:2009) by NRM Laboratories (Bracknell, UK). First the soils were tested for CaCO_3_ (which can cause an over-estimation of clay content) by adding a few droplets of 10% HCl, and CaCO_3_ was found in some of the European soils but none above 10% (where otherwise the texture results would be viewed with caution). Soils were also pre-treated to remove organic carbon (OC) from all samples: 30 ml of 30% volume hydrogen peroxide (H_2_O_2_) solution was added to soil and heated on a hotplate to 80 °C, this was repeated until fizzing had abated. Soils were then rinsed with deionised water and centrifuged to remove the clear supernatant, then re-dried and sieved to < 2 mm. Texture was classed as: clay < 2 µm, silt 2–63 µm, sand > 63–2000 µm. Note that in the Broadbalk independent validation set, sand was calculated as silt + sand, because traditionally a different sieve-pipette classification had been used^19^.

### Texture analysis using laser diffraction (LDA)

Of each of the soil samples, two replicate samples of approximately 1–3 g of < 2 mm soil (increasing with the transparency of the sample i.e. the quantity of sand), not pre-treated for OC removal, were analysed with LDA. Reference samples of an in-house standard soil were included in each run for quality control. A Partica LA-960 (Horiba Ltd, Kyoto, Japan) was used. This measures particles from 0.01–3000 µm with 93 particle size bins, using a diode laser of 650 nm wavelength and a blue LED light source of 405 nm wavelength. In the wet mode, the water fill level was 300 ml. A de-gassing procedure of the water bath is first conducted followed by a blank solution test to check for 100% light transmittance before soil is added. To aid with soil dis-aggregation, a 2 ml 4% Calgon (sodium hexametaphosphate) solution was added to the water bath immediately prior to adding the soil (ISO 11277:2009). Soil was then added to the water bath ensuring that the light transmittance/obscuration stayed between the thresholds 90–80% for red light and 90–70% for blue light. A re-circulation system with an ultra-sonic probe, flow cell and a centrifugal circulation pump stirs, disperses and pumps the water bath at 10 revolutions/s. The auto-mode refractive index was used (1.6 for standard polystyrene latex spheres). Ultra-sonification was applied with a power of 7 and ran at the start of each loop for 1 min. The suspension was then pumped through a sample cell placed in the convergent laser beam. There were 4 loops per sample, each loop ran for ~ 3 min, adding to a total of 12 min per sample. Particle size was calculated on a volume basis using the Mie theory with the proprietary software (Mie theory takes into account light transmission through the particle and yields a better prediction of high-angle scattering by small particles). A mean of the 4 loops of each replicate, and then of the 2 replicates was calculated. Texture was classed as: clay < 8 µm, silt 8–63 µm, sand > 63–2000 µm. This higher clay threshold compared to that of the sieve-pipette technique was used because it has been reported previously to give an improved estimate of clay with the LDA^5,11^. Note that two samples, one with high clay (58%) and one with high OC (33%) could not be analysed with the LDA because they had obscuration outside of the set threshold when added to the water bath.

### Texture analysis using mid-infrared (MIRS)

Of each of the soil samples, two replicate samples of approximately 0.5 g each of ground soil, which were both pre-treated and not pre-treated for OC removal, were scanned. Reference samples of an in-house standard soil, and blanks, were measured in each run for quality control. Analysis was conducted with a TENSOR II benchtop FT-IR (Fourier-Transform Infrared) spectrometer (Bruker, Berlin, Germany). This has a spectral range of 8000–340 cm^−1^, a KBr broadband beam-splitter and window, and an MCT (mercury cadmium telluride) mid-band detector cooled by liquid nitrogen. Diffuse Reflectance Infrared Fourier Transform (DRIFT) spectra were collected with a diffuse reflectance accessory. A background spectrum was taken with a gold-plated reference cap. The high throughput screening accessory (HTS-XT), which scans 95 samples in one plate, was used. The spectral resolution was 4 cm^−1^ and scan time was 32 s per sample. Absorbance data in the spectral range 4000–600 cm^−1^ were obtained.

Corrections of the raw data were made using the first derivative, with 8 smoothing points using the Savitsky–Golay algorithm. The scattering component resembles a very broad absorbance band, and first derivative correction suppresses broad bands relative to sharp bands, as well as eliminating baseline shifts. However, because a decrease in signal-to-noise ratio can result from first derivative correction, smoothing with the Savitzky–Golay polynomial technique is used to counteract this. Data was also corrected using the z-score: z = (x − μ)/σ. A comparison of the data correction methods showed that the first derivative as compared to the z-score gave slightly better performance, so first derivate corrected data was used throughout. CO_2_ peaks at 2361 and 2339 cm^−1^ were removed from the data. An average of every 10 wavenumbers was calculated from the data, which reduces the effects of high collinearity in multi-variate analysis, resulting in 236 wavenumbers (latent factors) in the model.

Mid-infrared calibration models were built from the sieve-pipette data for clay and sand using PLS (partial least-squares) modelling, with up to 20-fold LOO (leave-one-out) cross-validation (CV). The optimal number of components for the calibration models were selected based on the LOO CV which gave the lowest RMSE, this reduces the potential for over-fitting. Assessments of model predictive performance were made with validation set calculations of the Pearson’s correlation coefficient (R^2^, a measure of relative precision and closeness to the line of best fit) and intercept, RMSE (a measure of absolute accuracy and closeness to the one to one line), and the RPD (SD of observed values/RMSE of predicted values, which allows comparison of model performance across different data sets). The predictive performance was classified as: good if R^2^ > 0.75 (RPD > 2), average if R^2^ 0.75–0.5 (RPD 1.4-2), and poor if R^2^ < 0.5 (RPD < 1.4)^20^. The wavenumbers with the highest coefficient explaining the most variation in texture were identified. Statistical analyses and modelling with the pls package^21^ were performed in the R environment (R Core Team, 2016).

## Results

### Comparison of LDA and MIRS texture measurements

Originally there were 78 soils in the European calibration set and 21 in the Kenyan set, but 3 and 5 high clay outliers (clay > 60%) respectively, were removed from the data because of adverse effects on the clay calibration (discussed further below). Furthermore, in order to examine the effects of OC on texture measurements, the European set was split into sub-sets with < 5% OC (the majority with < 3% OC, n = 57) and > 5% OC (n = 13), and the soil set with < 5% OC was further randomly split into a calibration set (65%, n = 36) and a validation set (35%, n = 21), and the calibration sub-set was then used to predict clay in the < 5% OC and > 5% OC validation sets separately. The European whole calibration set (n = 75) was used to predict the European independent validation sets.

**Figure 1:**
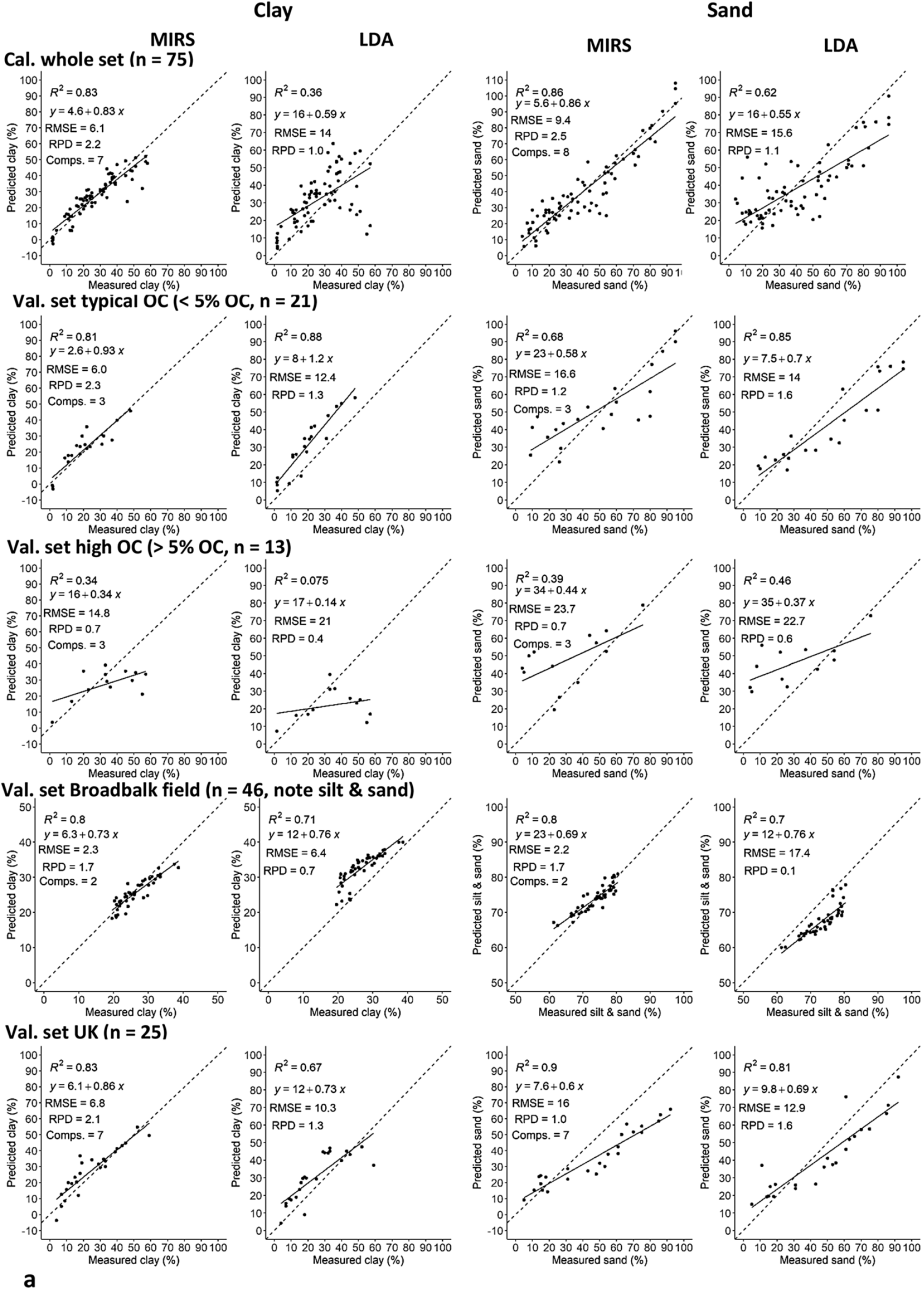

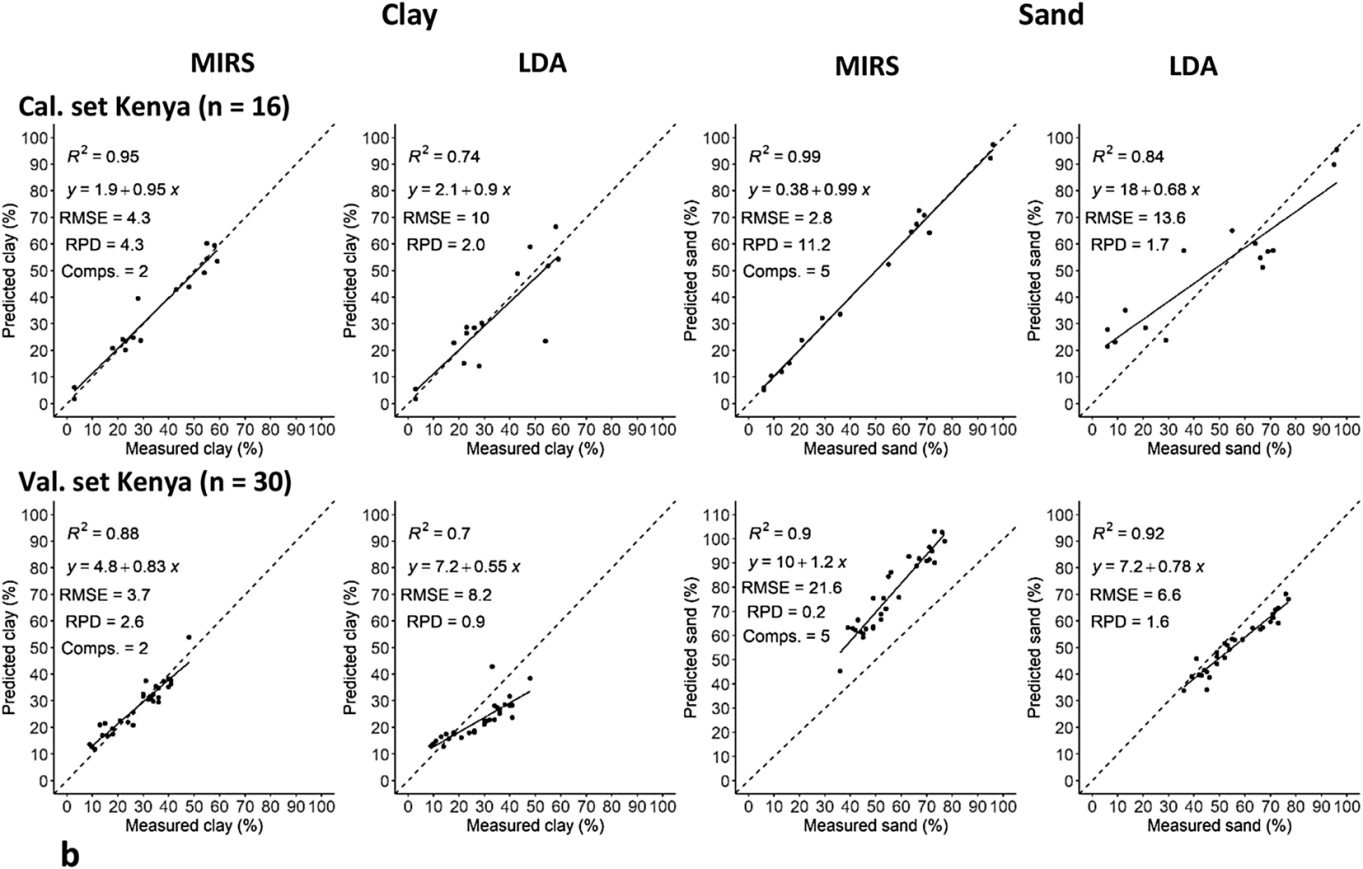
Measured (sieve-pipette) and MIRS and LDA predicted clay and sand in (**a**) the European whole calibration set (n = 75), European validation sub-set with < 5% OC (n = 21), European validation sub-set with > 5% OC (n = 13), Broadbalk independent validation set (n = 46, note sand & silt) and the UK independent validation set (n = 25) and (**b**) the Kenyan calibration set (n = 16) and the Kenya farm independent validation set (n = 30). Showing the R^2^, intercept, RMSE, RPD (standard deviation of observed values/RMSE of predicted values), and number of MIRS model components. Dashed line is the 1:1 line. Cal. = calibration, val. = validation. Note, LDA clay threshold < 8 µm.

The correlations between the LDA predicted values and the sieve-pipette measurements, and the MIRS predicted and the sieve-pipette measurements of clay in the European sets were, respectively; R^2^ = 0.36 and 0.83 in the whole calibration set; R^2^ = 0.65 and 0.67 in the calibration sub-set (3 components, data not shown, available in raw Supplementary Information, Appendix A); R^2^ = 0.88 and 0.81, in the validation sub-set with < 5% OC; R^2^ =  < 0.10 and 0.34 in the validation sub-set with > 5% OC; R^2^ = 0.71 and 0.80 in the Broadbalk set, and R^2^ = 0.67 and 0.83 in the UK set. For sand, the correlations were, respectively; R^2^ = 0.62 and 0.86 in the whole calibration set; R^2^ = 0.72 and 0.67 in the calibration sub-set (3 components, data not shown, available in raw Supplementary Information, Appendix A); R^2^ = 0.85 and 0.68 in the validation sub-set with < 5% OC; R^2^ = 0.46 and 0.39 in the validation sub-set with > 5% OC; R^2^ = 0.70 and 0.80 in the Broadbalk set, and R^2^ = 0.81 and 0.90 in the UK set. The RPD of the LDA predictions of clay were poor ranging from 0.4–1.3, and with the MIRS were poor to good ranging from 0.7–2.3. The RPD of the LDA predictions of sand were poor to average ranging from 0.1–1.6, and with the MIRS were poor–good ranging from 0.7–2.5 (Fig. 1a).

In the Kenyan sets, the correlation between the sieve-pipette measurements and the LDA predictions, and the sieve-pipette measurements and MIRS predictions of clay were, respectively; R^2^ = 0.74 and 0.95 in the calibration set, and R^2^ = 0.70 and 0.88 in the Kenya farm validation set. For sand, the correlations were, respectively; R^2^ = 0.84 and 0.99 in the calibration set, and R^2^ = 0.92 and 0.90 in the Kenya farm validation set. The RPD of the LDA predictions of clay in the Kenyan sets were poor–good from 0.9 to 2.0, and with MIRS were good from 2.6 to 4.3. The RPD of the LDA predictions of sand were average from 1.6 to 1.7, and with the MIRS were good in the calibration set at 11 but very poor in the validation set at < 1 (Fig. 1b).

Therefore, in both the European and Kenyan sets, clay predictions with MIRS were mostly better than the values given by the LDA. However, the LDA gave good measurements of sand in general, and gave a better prediction than the MIRS of sand in the Kenyan validation set. The LDA also gave better predictions than MIRS of both clay and sand in the European validation sub-set with < 5% OC. Furthermore, both methods gave very poor predictions of the high OC (> 5% OC) sub-set, and they both generally better predicted the Kenyan compared to the European soils.

**Figure 2:**
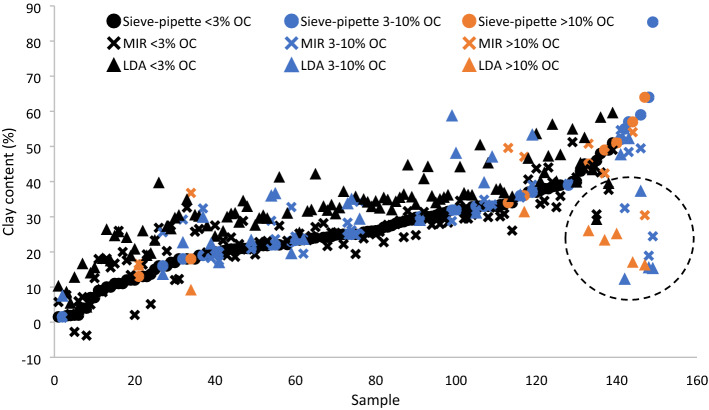
Clay content (%) in all samples of the European sets (including the calibration sub-set, validation sub-sets < and > 5% OC, independent validation sets, and high clay outliers > 60% clay, n = 144), measured by sieve-pipette (filled circle), MIRS (crossed) and LDA (Filled triangle, at < 8 µm particle size threshold). The sets have also been categorised by OC content: < 3% (black), 3–10% (blue), > 10% (orange). The dashed circle shows the LDA and MIRS predictions of samples with high clay and > 10% OC grouping below the sieve-pipette values.

The absolute accuracy of LDA (triangles) and MIRS (stars) values of clay compared to the sieve-pipette measurements (circles) were compared in all European sets. The LDA generally over-estimated all clay contents by on average ~ 60%, but under-estimated high clay contents by ~ 60% (with a particle size threshold of < 8 µm). The MIRS predictions of clay were generally very close to the sieve-pipette measurements, but with on average a ~ 60% over-estimate at very low clay contents and ~ 10% under-estimate at high clay content. However, both techniques had a much bigger under-estimate of clay of > 300% in the high clay outlier samples (those with > 60% clay content) (Fig. 2).

The effect of OC on clay detection in the European sets was also analysed in samples with < 3% OC (black), 3–10% OC (blue), and > 10% OC (orange). In samples with < 3% OC content the LDA over-estimated clay content by on average ~ 30% and the MIRS gave very accurate predictions. In samples with 3–10% OC content the LDA and MIRS predictions of clay were variable- sometimes over and other times under-estimating clay content. In samples with > 10% OC content the LDA under estimated clay content by on average ~ 80%, whereas often the MIRS over estimated clay content by on average ~ 15%. Interestingly, it can also be seen that often the soils with higher clay content also had high OC content, and both the LDA and the MIRS gave significant under-estimations of clay in these soils (indicated by dashed circle) (Fig. 2).

**Figure 3:**
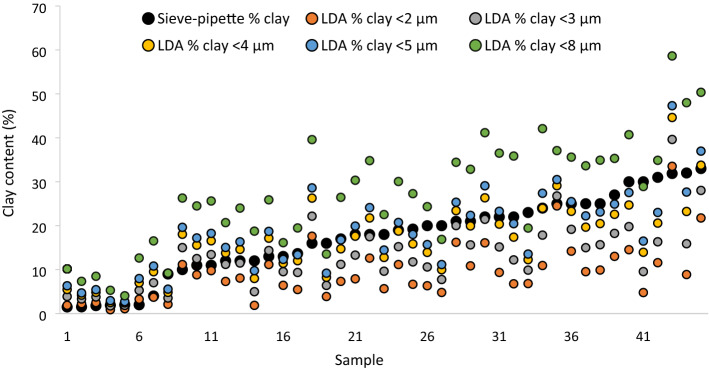
Clay content (%) in the European soil set with < 3% OC content (n = 45) measured by LDA at particle size thresholds of: < 2 (orange circle), < 3 (grey circle), < 4 (yellow circle), < 5 (blue circle) and < 8 (green circle) µm and compared to the standard sieve-pipette measured values (filled circle).

The LDA threshold of clay particle size was < 8 µm, and not < 2 µm as in the sieve-pipette measurements, and with this, clay was over-estimated by ~ 60%. Therefore, LDA clay thresholds of < 2, < 3, < 4, < 5 and < 8 µm were compared to find the most reliable threshold in the < 3% OC set (which the LDA had predicted with the best precision). At < 2 µm the clay content was under-estimated by on average 90% (R^2^ = 0.68); at < 3 µm clay content was under-estimated by on average 30% (R^2^ = 0.74); at < 4 µm clay content was under-estimated by on average < 1% (R^2^ = 0.76); at < 5 µm clay content was over-estimated by on average 6% (R^2^ = 0.77), and at < 8 µm clay content was over-estimated by on average 70% (R^2^ = 0.78). Therefore, the absolute accuracy of the clay prediction was best at the < 4 µm particle size threshold (Fig. 3).

### MIRS texture analysis

As mentioned, samples with very high clay content (> 60%) were first removed from the European (n = 3) and Kenyan (n = 5) MIRS calibration sets, because clay content in these samples was significantly under-estimated by MIRS (Fig. 2), and when included they generally had an adverse effect on the clay prediction causing it to be over-estimated. For example, in the European calibration sub-set, the prediction of clay with high clay samples included versus removed gave an RMSE of 13 and 11 respectively, and in the validation set with < 5% OC R^2^ = 0.27 and R^2^ = 0.80, respectively. In the Kenyan calibration set, predicting clay with high clay samples included versus removed gave an RMSE of 11 and 6 respectively, and an R^2^ of 0.82 and 0.95, respectively (data not shown, available in raw Supplementary Information, Appendix A). Corroborating this finding that extremes of texture were not detected well by MIRS, it can also be seen in both the European and Kenyan soil sets that the regression line tilts to a flatter slope than the one to one line in both the clay and sand predictions; therefore small clay/sand contents are generally over-estimated and high clay/sand contents are generally under-estimated (Fig. 1).

### MIRS texture analysis in soils with and without OC

**Figure 4:**
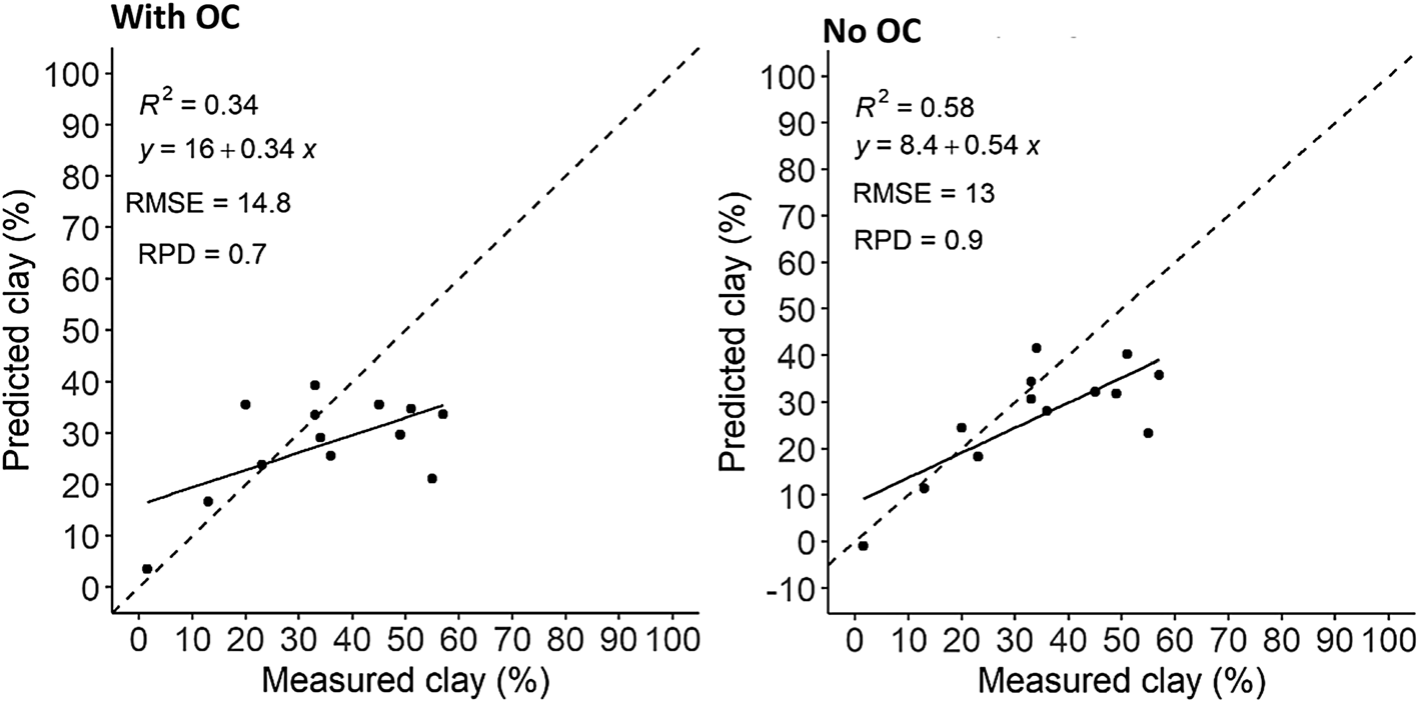
Clay content measured (sieve-pipette) and predicted by the MIRS calibration models with OC and with OC removed from the soils in the European validation subset with > 5% OC (n = 13). Showing the R^2^, intercept, RMSE and RPD (standard deviation of observed values/RMSE of predicted values). Dashed line is the 1:1 line.

In the European set, MIRS predictions of clay were only average and tended to be over-estimated in soils with higher OC (> 5% OC), and under-estimated in high clay soils (> 60% clay) with very high OC (> 10% OC) (Fig. 2). It was therefore of interest to remove OC from the soils to see if the predictions improved. In the validation sub-set with > 5% OC there was a reduction in the over-estimation of lower clay contents and a reduction in the under-estimation of high clay contents; the intercept decreased from 16 to 8, the RMSE decreased from 15 to 13, and the precision increased from R^2^ = 0.34 to R^2^ = 0.58 (Fig. 4).

**Figure 5:**
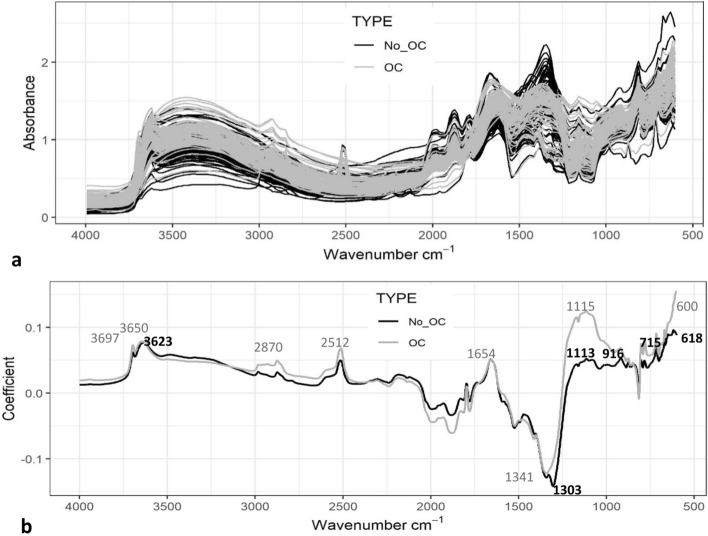
MIRS analysis of clay content in the European calibration set: (**a**) raw spectra and (**b**) raw spectral coefficients of component 1, in soils with OC removed (black line) and with OC (grey line).

Figure 5a shows the raw MIRS spectra of the European whole calibration set in soils with and without OC. A qualitative analysis of the spectra in the soils with OC removed shows that there are no broad peaks at ~ 2900–2800 cm^−1^, and there is a reduction in peak intensity at 1115 cm^−1^. Figure 5b shows the coefficients explaining clay content in the soils with and without OC, these still correspond closely at 3697/3650/3623 < 2512 and 1654 cm^−1^, except for a distinct reduction in the peak intensity at 1115 cm^−1^.

### European and Kenyan texture spectral coefficients

**Figure 6:**
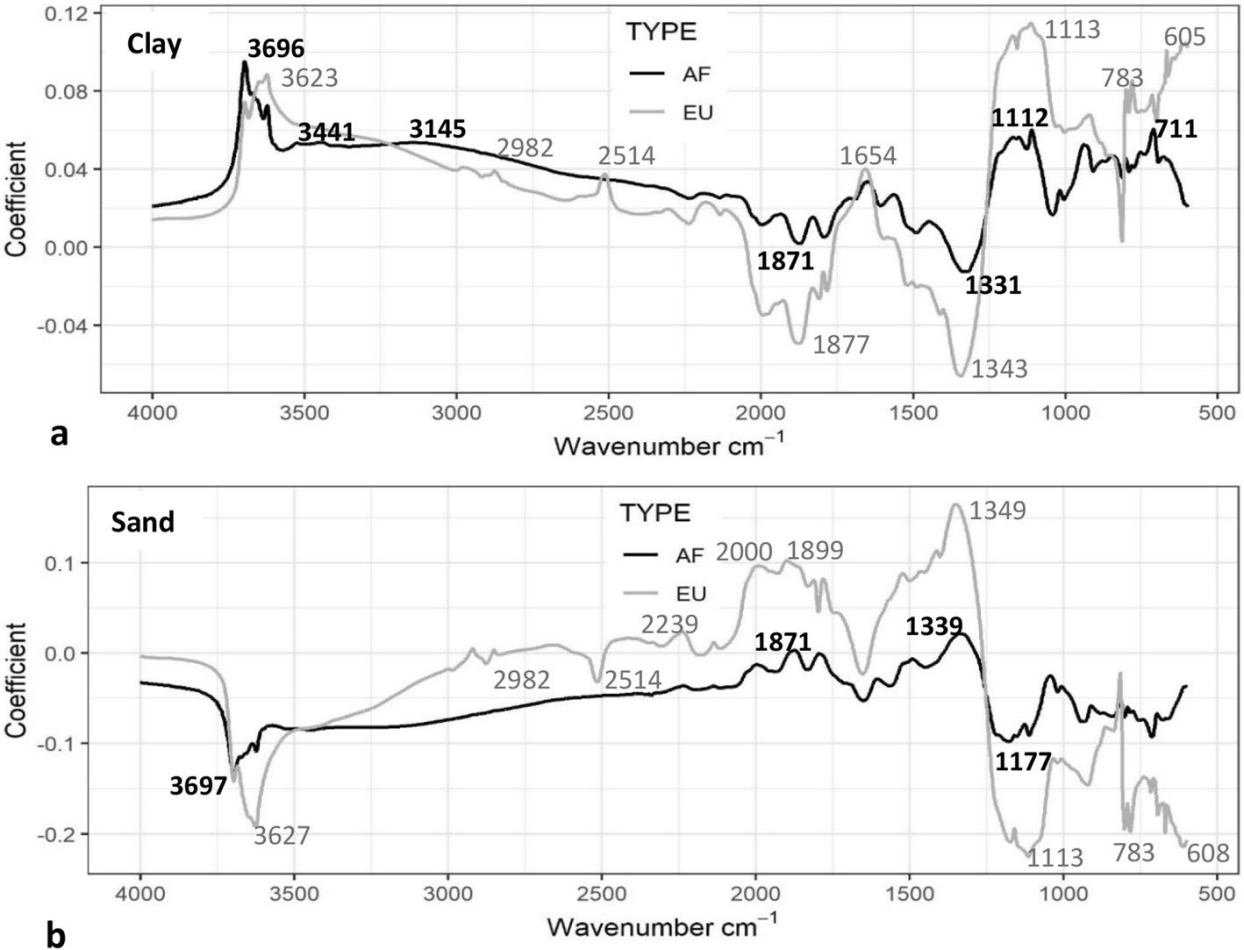
MIRS calibration model component 1 raw spectral coefficients predicting (**a**) clay and (**b**) sand content in the European (grey line, n = 75) and Kenyan (AF, black line, n = 16) calibration soil sets.

In the European calibration set, the coefficients explaining the most variation in clay content were at spectral regions: 1113 < 605 < 3623 < 783 < 2982 < 1654 < 2514 cm^−1^ (Fig. 6a), and 1349 < 1899 < 2000 < 2239 cm^−1^ for sand content (Fig. 6b). In the Kenyan calibration set, the coefficients explaining the most variation in clay were at spectral regions: 3696 < 1112 < 711 < 3145 < 3441 cm^−1^ (Fig. 6a), and in sand were 1339 < 1871 cm^−1^ (Fig. 6b). Thus, there were overlaps but also differences between the European and Kenyan sets in the spectral regions explaining the most variation in clay; particularly in the peak intensity at 1113 cm^−1^, and a slight peak shift from 3623 cm^−1^ in the European set to 3696 cm^−1^ in the Kenyan set, and in the Kenyan set there were no peaks at 2982, 1654 and 2982 cm^−1^ as there were in the European spectra. The spectral regions predicting sand were the inverse to those of clay.

## Discussion

Compared with the sieve-pipette method, the precision in detection of the clay fraction with the LDA was poor—average in most soil sets, but very good in the European set soils with < 5% OC (Fig. 1). Similarly poor correlations between LDA and conventional measurements due to clay under-estimation have been observed previously; R^2^ of 0.74^2^, R^2^ = 0.70^3^ and R^2^ = 0.16^4^. In terms of the absolute accuracy of LDA measurements, clay was over-estimated by ~ 60% in most soils and under-estimated by ~ 60% in very high clay soils, with a < 8 µm threshold (Fig. 2), but it was under-estimated by ~ 90% with a < 2 µm threshold (Fig. 3). However, in the present study when the clay threshold was set at < 4 µm in the soils with < 3% OC there was very good precision and accuracy (Fig. 3). This confirms previous studies which found that LDA gave an under-estimate of clay with a clay threshold < 2 µm ^4,5,7,22,23^, but disputes the proposed clay threshold of < 8 µm with LDA analyses^5,11^. Taubner et al.^6^ also found an ~ 20% clay over-estimate using the < 8 µm threshold. A part of this discrepancy could be due to the fact that the corrective threshold is dependent on the particle size distribution just above 2 µm. If there are many particles in that range, the correct threshold value will be close to 2 µm, if there are few particles in that range the correct threshold will be higher.

An additional explanation for this discrepancy in findings on the ideal clay threshold could be related to the pre-treatment of samples to remove OC in the afore-mentioned LDA studies^4,5,7,22^. In the present study, the absolute accuracy of the clay estimate with the LDA was very good for soils with < 3% OC and a < 4 µm threshold, even without OC removal (Fig. 3). It may be that using oxidising agents to remove OC disintegrated/destroyed expandable clay minerals^24^, thereby actually reducing the clay content. Hydrogen peroxide is a weak acid, and when exposed to acids the structure of many clays is destroyed, and they are dissolved to finely dispersed amorphous silica or silica oxides^25^. Silica is more transparent than clay, and as well as the small size this may make them more difficult to detect with the LDA. Conversely, the clay particles may have increased to a size above the < 2 µm threshold, as clay minerals are expandable, and H_2_O_2_ and high temperature have been shown to increase the size of clay particles by 80–120-fold^26^. Both processes would necessitate a higher clay threshold to increase the detection of the clay proportion. Mikutta et al.^27^ suggest that pre-treatment with H_2_O_2_ should be avoided for the determination of mineral phases. It should also be kept in mind that the sieve-pipette technique can over-estimate the clay proportion^3,4^. Likewise, Fisher et al.^22^ found no significant effects of OC removal on clay detection in soils which generally had < 5% OC, and the only effects were seen in podsol soils from pasture systems with large contents of particulate labile carbon. Beuselinck et al.^8^ found no significant effect of OC removal on clay detection. Di Stefano et al.^7^ found only a very minimal effect of H_2_O_2_ treatment in soils with < 3% OC. Yang et al.^4^ even reported a significant under-estimate of clay even with OC removal. However, Vdovic et al.^2^ found a significant increase in clay detection in soils with OC removed in soils with > 5% OC content. Thus perhaps it is particulate carbon in soils with particularly high OC content which shifts the estimate with LDA to larger particle fractions, and only such soils require OC removal for accurate clay measurement. In summary, the results show that for good estimates of clay with the LDA in typical soils with < 3% OC, pre-treatment was not necessary, and the clay threshold needed to be increased only slightly to < 4 µm.

The LDA sand estimation was better than the clay estimate (Fig. 1), as previously observed^2–4,6,8^. These studies indicated that the clay under-estimate was compensated in the silt proportion rather than in the sand proportion, and therefore the sand estimate was un-affected^3,4,6,8^. This is also in agreement with previous findings that the assumptions of sphericity made by the sieve-pipette and LDA methods are more applicable to sand particles^6–8^.

With both the LDA and MIRS, the clay predictions were better for the Kenyan compared to the European soils (Fig. 1). This could be due to lower OC in the Kenyan sets (max 6% in all soils, Table 1), as discussed above. Additionally, there was a greater diversity of soil types and range in the soil properties in the European calibration set, which came from 11 different countries (Table 1). It has been observed with the LDA that separating by soil type e.g. in soils rich in oxides or organic matter, improves estimates, because different soil types may require different technical settings and data transformations such as in the refractive index^3,6,7^. Likewise with MIRS, greater sample diversity generally leads to higher prediction errors, and restricting the calibration set to samples similar to the set to be predicted improves predictions^17^. Predictions of soil texture with MIRS in samples restricted to a very small geographical range give very good predictions^13,15,18^.

Mid-infrared spectroscopy is known to give texture predictions consistent with the sieve-pipette technique^13–18^. A clear advantage of the MIRS technique is that it analyses the chemistry of soil constituents and is not affected by particle shape effects. The results show that MIRS predictions of clay and sand content were very close to the sieve-pipette measurements in this very wide range of soils and textures. But although the precision of texture predictions was very good, small clay/sand contents were generally over-estimated and high clay/sand contents were generally under-estimated compared with the sieve-pipette method (Figs. 1 and 2), suggesting that extremes of texture content were not adequately detected by MIRS. The same trend of sand and clay over-estimation at low contents, and under-estimation at high contents, was observed previously^28^. Probably as a consequence of this trend, the MIRS predictions were significantly improved with very high clay samples (> 60%) removed from the calibration set. These errors might also have been caused by our relatively small calibration set with a limited number of samples in the extremes of clay content.

There were overlaps but also differences between the European and Kenyan sets in the spectral coefficients explaining the variation in clay; particularly in much greater peak intensity at 1112/1113 cm^−1^ in the European soils (Fig. 6). This peak has previously been found to relate to Si–O stretching in clays^29^ and the sorption of organic matter to the surfaces^30^, discussed further below. There was also a slight peak shift from 3623 cm^−1^ relating to smectite and illite^18,28,31^ in the European set, to 3696 cm^−1^ relating to kaolinite clay and Fe oxides^15,28,29^ in the Kenyan set. In the European set there were peaks at 2982–2870, 2512 and 1654 cm^−1^ which were not observed in the Kenyan set. These relate to OM^16,31,32^, carbonates^16^, and H–O–H bonds of water in the clay lattice^33^, respectively. Many types of clay have strong absorbance bands in the same region as organic matter, and this prevents the accurate detection of these over-lapping properties^32^. It is likely therefore that the peaks for OC and carbonates observed in the coefficients predicting clay in the European soils over-lapped with clay peaks, and this interfered with an accurate detection of clay. Whereas, only peaks relating to clay were observed in the Kenyan soils, explaining the more accurate detection of clay in these soils.

The MIRS prediction of clay in soils with OC removed improved in accuracy and precision in the soils with > 5% OC (Fig. 4). The coefficients explaining clay content in the soils with and without OC removed corresponded closely, except for a distinct reduction in peak intensity at 1113/1115 cm^−1^, and a much smaller reduction in peak intensity at ~ 2900–2800 cm^−1^ (Fig. 5). The broad peaks at ~ 2900–2800 cm^−1^ were identifying C–H bands in aliphatic compounds/OC^16,31^. Reeves et al.^32^ observed that with ashing of soils to remove OC, the region 3000–2800 cm^−1^ did not alter the spectra of clay, indicating that no spectral over-lap with OC and clay occurred in this region, as observed here. The peak at 1113/1115 cm^−1^ has previously been identified as relating to Si–O stretching in kaolinite and montmorillonite and amorphous silica^29,30,34^, and more specifically the sorption of organic matter^30^, humic^35–37^ and fulvic^38^ acids on to the surfaces of these clays. This suggests that the intensity of the peak at 1115 cm^−1^ in the clay model coefficients in the soils with OC was enhanced by the OC associated with the clay, and with this peak no longer so predominant in the coefficients in soils with OC removed, the prediction improved. Interestingly, as mentioned above, this peak at 1113 cm^−1^ was also much less pronounced in the coefficients explaining clay in the Kenyan compared to the European set.

## Conclusion

Clay measurements were generally much better with MIRS than the LDA, in both precision and accuracy. Although, the LDA estimate of sand was good. Unlike the LDA, MIRS analysis is not subject to the limitations imposed by the shape and density of particles, which most likely explains the improved predictions. However, the LDA would be a good technique in soils with typical levels of OC found in agricultural soils, as observed here in the sample sub-set with < 5% OC content. Accuracy in prediction of clay with the LDA was best with a clay threshold of < 4 rather than < 8 µm. The predictions of clay with both the LDA and MIRS were poor in soils with higher OC, in the case of the MIRS this was caused by interference between overlapping peaks for OC and mineral constituents. With both the LDA and MIRS, separating soils into sets with very different OC and clay contents would improve predictions; enabling different refractive index settings to be used with the LDA, and separate calibrations to be made with the MIRS. It is concluded that both techniques could be used for cheap, fast and reliable estimates of soil texture in typical agricultural soils with < 5% OC and < 60% clay content.

## Supplementary information

Supplementary Information 1.

## Abbreviations

MIRS: Mid-infrared spectroscopy
LDA: Laser diffraction analysis
OC: Organic carbon
